# Analytic Study

**Published:** 1888-09

**Authors:** Edward Fornias

**Affiliations:** 711 Pine Street


- **PARESIS or PARALYSIS, motor. (Single nerve.)**
  - *Face.*—Semilateral, with sensation of tightness and pain in the jaws, rendering very difficult to open the mouth or to eat.
  - *Tongue.*—Stuttering, indistinct speech; speechlessness.
  - *Upper eyelid* (oculo-motor nerve).—Ptosis.
  - *Larynx--Aphonia*—Weak voice (in singers and speakers). The muscles refuse to act on account of utter weakness. The voice cannot be raised; it fails, or become a squeak.
  - *Bladder* (sphincter).—From long retention. In children who pass urine unconsciously during first sleep. *Dribbling of urine while sneezing, blowing the nose, or coughing.* (Thuja.)
  - *Rectum* (muscular fibres). Inactivity, from weakness; ineffectual urgings, with much pain, anxiety, and redness of face; only while standing is able to pass the stool. (Verified.)
  - *One side of body* (hemiplagia).—After cerebral hemorrhage or softening; right or left side; tension and softening of muscles; dull drawing, or bruised pain in the coccygeal region.
  - *Deltoid.*—Cannot raise hand to head.
- *Epilepsia.*—Recent cases. During time of puberty; < during new moon; cold water brings the paroxysm on again; he drenches himself with urine during the spasm.
- *Chorea.*—Convulsive movements, with distortion of the eyes; right side of face and tongue may be paralyzed.
- *Convulsions.*—With screams, gnashing of teeth, and violent movements of the body. During the attack the urine flows copiously and involuntary.
- *Rheumatism and Arthritis.*—Contraction of flexors and stiffness of joints (Guayac?; in the articulation of the jaws; numbness of the parts; pains drawing and tearing; at night cannot lie still a minute; part laid on feels bruised (Arn., Bapt.); paralytic weakness, and trembling of limbs; sour-smelling night-sweats (Sil.). *Arthritis deformans.*

| | |
|---|---|
| CATARRHS, *Laryngeo-tracheal* | *Hoarseness.*—Scraping; rawness of throat and chest.<br>*Aphonia.*—Sudden loss of voice on taking cold; cannot speak a loud word.<br>*Cough.*—dry, hoarse, with adhering mucus in chest, > by a swallow of cold water; no power to hawk up or spit out the phlegm (paresis); *every fit of cough causes dribbling of urine;* burning soreness in a stripe down under the sternum. |
| COLIC, | *Flatulent or Menstrual.*—Must bend double (Coloc.) < after the least food, or from tight clothes; painful distention; loud rumbling. |
| WARTS, | *Location.*—Face, nose, eyelids, arms.<br>*Nature.*—Old; large; inflamed; ulcerated; painful. |

In the synopsis of *Causticum*, given above, I have endeavored to embrace all that every student of materia medica should know about this antipsoric in order to make an appropriate use of it, and, although its therapeutical individuality rests principally upon the knowledge of this summary, a few words on its general action will undoubtedly serve to better understand and retain its distinctive feature.

*Causticum Hahnemanni* (not Kali Causticum) is a drug the exact constituents of which are not known. Dr. Black thinks it is a weak solution of *potassic hydrate.* But, though deficient as to the knowledge of its nature, its value as a remedy has been well established by clinical experience. In the high potencies especially it has done a mighty work, by bringing relief to many a victim of disease.

So as in *Arsenicum* we look for the *irritability of fibre* as a prime indication. In *Causticum* it is the *paralytic weakness* which furnishes us the leading characteristic, showing plainly the origin of the drug. True enough, Causticum, like Arsenicum and Rhus tox., exhibits marked signs of restlessness, but these are usually present in rheumatic and arthritic affections and invariably occur at night, giving no positive relief. On the other hand, the *paralytic weakness* seems to be an almost constant expression of Causticum. Even in the lower bowels we find evidences of this weakness, for there is fecal accumulation, with inability to expel it. The efforts producing (especially in children) much pain, anxiety, and redness of the face. And, as Dr. Farrington so well observed, "*this paresis is further illustrated in scrofulous children, who totter and fall while walking.*"

Now, if in addition to this we study the Causticum patient, we cannot fail to form a correct estimate of the use of this important drug. *Fear* is the leading mental condition, but *melancholy, sorrow, weeping, vexation, irritability of mind,* and *anger, with scolding,* are also phenomena of Causticum. This fear is not the fear of death of *Aconite* or *Arsenicum,* nor the fear of losing the senses of *Calc. ost.,* but *an apprehension of evil,* day and night, which makes the patient anxious and low-spirited. She is especially *afraid at night in the dark,* and if a child, does not want to go to bed alone. When closing the eyes terrible images and distorted human faces appear before him. The *melancholy* and *sorrow* leave a marked impress upon the face ; the former is such that she looks on the dark side of everything, the latter is usually attended by weeping, reminding us of *Pulsatilla.* Vexed, irritable mood, and attacks of anger, with scolding, are likewise conditions indicative of *Causticum.*

As indications of *Causticum* we must also remember—that injuries which had healed become sore again—that during lactancy the milk often disappears in consequence of over-fatigue, night-watching, care, or anxiety ;—that during the menses no blood is passed at night (Bovista only at night), and after their cessation a little blood is passed from time to time for many days, which smells bad,—and that *Causticum* is especially suitable to subjects with dark hair and rigid fibre, and to children with delicate skin.

In regard to *relationship* with other drugs we find *Causticum* antidoted by *Coffea, Coloc., Spir. nitr. dulc.,* and *Nux vom.*—frequently suitable before *Sepia* and *Stann.*—and bearing an inimical or antagonistic relation to *Phosph.* In colics *Causticum* follows *Coloc.,* acting as its complementary.

711 Pine Street. EDWARD FORNIAS.

